# *Mycobacterium tuberculosis* Rv0928 protein facilitates macrophage control of mycobacterium infection by promoting mitochondrial intrinsic apoptosis and ROS-mediated inflammation

**DOI:** 10.3389/fmicb.2023.1291358

**Published:** 2023-10-31

**Authors:** Chenling Xu, Yan Yue, Sidong Xiong

**Affiliations:** Jiangsu Key Laboratory of Infection and Immunity, Institutes of Biology and Medical Sciences, Soochow University, Suzhou, China

**Keywords:** *Mycobacterium tuberculosis*, Rv0928, SLC25A3, mitochondrion, apoptosis, inflammation

## Abstract

Macrophages are the main target cells for *Mycobacterium tuberculosis* (Mtb) infection. Previous studies have shown that Mtb actively upregulates phosphorus transport proteins, such as Rv0928 protein (also known as PstS3), to increase inorganic phosphate uptake and promote their survival under low phosphorus culture conditions *in vitro*. However, it is unclear whether this upregulation of PstS3 affects the intracellular survival of Mtb, as the latter is also largely dependent on the immune response of infected macrophages. By using Rv0928-overexpressing *Mycobacterium smegmatis* (Ms::Rv0928), we unexpectedly found that Rv0928 not only increased apoptosis, but also augmented the inflammatory response of infected macrophages. These enhanced cellular defense mechanisms ultimately led to a dramatic reduction in intracellular bacterial load. By investigating the underlying mechanisms, we found that Rv0928 interacted with the macrophage mitochondrial phosphate carrier protein SLC25A3, reduced mitochondrial membrane potential and caused mitochondrial cytochrome c release, which ultimately activated caspase-9-mediated intrinsic apoptosis. In addition, Rv0928 amplified macrophage mitochondrial ROS production, further enhancing pro-inflammatory cytokine production by promoting activation of NF-κB and MAPK pathways. Our study suggested that Mtb Rv0928 up-regulation enhanced the immune defense response of macrophages. These findings may help us to better understand the complex process of mutual adaptation and mutual regulation between Mtb and macrophages during infection.

## Introduction

Tuberculosis is a communicable disease caused by *Mycobacterium tuberculosis* (Mtb) infection, and remained the leading cause of death from a single infectious pathogen until the coronavirus (COVID-19) pandemic in 2019. According to World Health Organization (2022), the newly diagnosed cases and deaths of TB in 2021 achieved 6.4 and 1.6 million, higher than those in 2020. Even more worrying is the fact that the TB disease burden might further worsen due to continually damaging impact of COVID-19 (Hegarty et al., 2020). So far BCG is the only approved TB vaccine, but its protective efficacy varies greatly in distinct counties and populations (Nieuwenhuizen and Kaufmann, 2018; Singh et al., 2022). Therefore, it is urgent and necessary to elucidate the interaction mechanisms between Mtb and host cells and to identify potential targets for novel prophylactic and therapeutic strategies.

Macrophage engulfment is critical for mycobacterium to establish successful and persistent infection. Accordingly, Mtb has developed complex mechanisms to adapt to the delicate and complicated intracellular niche in macrophages (Rengarajan et al., 2005). As an essential nutrient for all living organisms and limited in the environment, inorganic phosphate (Pi) must be acquired by Mtb during infection. To manage phosphate uptake and bacillary survival, Mtb genome contains several genes encoding phosphate-specific transporter (Pst) system, which consists of four protein families: PstS to detect and bind Pi, PstA and PstC to form Pi transmembrane entry pores, and PstB to offer Pi transport energy (Lamarche et al., 2008; Soni et al., 2020). Three PstS proteins (PstS1-3) have been identified in Mtb and have been shown to enhance Mtb growth *in vitro* under phosphate-limited conditions by increasing Pi uptake, as demonstrated in previous studies (Peirs et al., 2005; Rifat et al., 2009; Vanzembergh et al., 2010). In particular, PstS3 (also known as Rv0928) is the most prominently induced protein in low-phosphorus environments (Vanzembergh et al., 2010), and is one of the genes essential for Mtb growth in macrophages (Rengarajan et al., 2005). However, the effect of Rv0928 on intracellular survival of Mtb remains uncertain, since the latter is also largely dependent on immune defense responses of infected macrophages. Previous studies have demonstrated that deficiency of PstS protein significantly reduced the mycobacterium replication in lung tissues of infected mice (Peirs et al., 2005), suggesting that apart from promoting phosphate uptake, Pst family might also impact host immune defenses.

In this study, we investigated the role of Rv0928 in mycobacterial infection of macrophages. We found that in response to infection with Rv0928-overexpressing *Mycobacterium smegmatis* (Ms::Rv0928), macrophages exhibited an enhanced immune response characterized by increased apoptosis, facilitated secretion of pro-inflammatory factors, and ultimately effectively limited intracellular bacterial survival. These findings suggest that upregulation of Rv0928, while facilitating the uptake of phosphorus atoms by mycobacterium, ultimately culminated in an enhanced macrophage immune response and consequently led to reduced intracellular bacterial replication.

By exploring the underlying mechanisms, we found that Rv0928 interacted with mitochondrial SLC25A3 in macrophages, facilitating membrane potential depolarization and mitochondrial cytochrome c release, which further promoted caspase-9-mediated intrinsic apoptosis. Meanwhile, Rv0928 also increased mitochondrial ROS production and enhanced NF-κB and MAPK (p38 and JNK) mediated pro-inflammatory pathways.

## Materials and methods

### Mice, cell lines, and mycobacterial strains

Six-to eight-week-old female C57BL/6 mice were purchased from the Laboratory Animal Center of Soochow University. The mice were housed in a barrier facility with controlled temperature at Soochow University. All animal experiments used in this study were approved by the Institutional Animal Care and Use Committees of Soochow University.

Murine RAW264.7 macrophage cell line, human HEK293T cell line were obtained from the American Type Culture Collection, and routinely cultured in RPMI 1,640 or DMEM supplemented with 10% fetal bovine serum (FBS) and penicillin (100 U/mL)/streptomycin (100 μg/mL) at 37°C with 5% CO_2_. Bone marrow derived macrophages (BMDMs) were prepared as previously reported (Zhang et al., 2008).

*Mycobacterium smegmatis* (Ms) mc^2^155 strain was used grown in Luria-Bertani medium supplemented with 0.05% Tween-80. The *M. tuberculosis* strain H37Rv were grown in Middlebrook 7H9 broth medium supplemented with 10% ADC, 0.5% glycerol, and 0.05% Tween-80 at 37°C.

### Construction of plasmids and recombinant *Mycobacterium smegmatis*

The Rv0928 gene was amplified from H37Rv genomic DNA by PCR and subsequently inserted into the eukaryotic expression vectors pCMV-HA or pFlag-CMV2 to generate pHA-Rv0928 or pFlag-Rv0928, respectively. PCR primers used to amplify genes of interest are listed in Table 1. These constructs were then used for transfection assays in HEK293T cells. In addition, the Rv0928 cDNA was inserted into the *E. coli*-Mycobacterium shuttle vector pMV261, and the resulting pMV261-Rv0928 and empty pMV261 plasmids were separately electroporated into the Ms. strain mc^2^155 to generate the Rv0928-overexpressing Ms. (Ms::Rv0928) and the control Ms.::Vector strain, respectively.

**Table 1:** Primers used in the study.

Primer sequence (5′–3′) for plasmid constructions	
pMV261-Rv0928	F: CCGGAATTCTTGAAACTCAACCGATTTGGTGC R: CAGAAGCTTTCACTTGTCGTCATCGTCTTTGTAGTC GGCGATCGCGTTGAC
pCMV-HA-Rv0928	F: CCGCGAATTCGGATGTTGAAACTCAACCGATTTGGTGC R: GGCTCTCGAGGT TCAGGCGATCGCGTTGAC
pFlag-Rv0928	F: CGCAAGCTTGCGATGTTGAAACTCAACCGATTTGGTGC R: GGCGGATCCTCAGGCGATCGCGTTGAC
pFlag-SLC25A3	F: GGCTCTCGAGGTATGTTCTCGTCCGTGGCG R: GCGGCCGCCTACTGAGTTAACCCAAGCTTCTTC
Plvx-IRES-ZsGreen-Rv0928	F: CCG GAATTC ATG GACTACAAAGACGATGACGACAAG TTGAAACTCAACCGATTTGGTG R: TGC TCTAGA TCAGGCGATCGCGTTGAC

Primer sequence (5′–3′) for real-time PCR analysis	
IL-6 (murine)	F: ACAACCACGGCCTTCCCTACTT
	R: CACGATTTCCCAGAGAACATGTG
GAPDH (murine)	F: GAGCCAAACGGGTCATCATCT R: GAGGGGCCATCCACAGTCTT
TNF-α (murine)	F: AAGCCTGTAGCCCACGTCGTA R: GGCACCACTAGTTGGTTGTCTTTG
MCP-1 (murine)	F: CCCACTCACCTGCTGCTACT R: TCTGGACCCATTCCTTCTTG

### Western blot assays

Cells were lysed in lysis buffer (10 mM Tris–HCl, pH 7.4, 5 mM EDTA, 150 mM NaCl, 1% Triton X-100, 1 mM PMSF, and protease inhibitor cocktail) for 30 min on ice, and then equal amount of lysate protein determined by a Bradford assay was separated by SDS-PAGE gels and transferred onto polyvinylidine difluoride membranes. After blocking with 5% fat-free milk for 2 h at room temperature, membranes were incubated indicated primary antibodies overnight at 4°C followed by incubation with HRP-conjugated goat anti-mouse or anti-rabbit secondary antibody (Abclonal Biotechnology). Membranes were finally developed with ECL Prime (New Cell and Molecular Biotech), and chemical luminesce signals were visualized using an Amersham Imager 600 (GE Healthcare).

Primary antibodies used for western blot assays including anti-β-tubulin, anti-β-actin, anti-caspase-3, 8, 9, anti-phosphorylated ERK1/2, anti-phosphorylated p65, anti-phosphorylated p38, anti-phosphorylated JNK, anti-cytochrome C, anti-GroEL (Cell Signaling Technology), anti-SLC25A3 (Santa Cruz Biotechnology), anti-Bax and anti-VDAC1 (Abclonal Biotechnology) antibodies. The mouse anti-Rv0928 serum polyclonal antibody was prepared by immunizing BALB/c mice with 25 μg of recombinant Rv0928 protein 3 times with 2-week intervals, sera were collected 2 weeks after the last immunization and used for western blot assays.

### ELISA assays

The levels of pro-inflammatory cytokine (TNF-α, IL-6, IL-1β, and MCP-1) in cell culture were measured using sandwich enzyme-linked immunosorbent assay (ELISA) kits according to the manufacturer’s instructions (Invitrogen).

### Flow cytometry

Apoptotic cells were detected using an Annexin V/7-AAD staining kit (BD Pharmingen) according to manufacturer’s instruction. For intracellular ROS level measurement, cells were then incubated with a cell-permeant ROS fluorescent probe, 2′, 7′-dichlorodihydrofluorescein diacetate (H_2_DCFDA, Invitrogen) for 30 min at room temperature in dark, and then subjected to flow cytometry on a FACS Canto II cytometer (BD Biosciences). To detect the Phagocytosis efficiency of macrophages, RAW264.7 cells infected with Ms.::Rv0928-mCherry or Ms.::Vector-mCherry for 2 h, and then subjected to flow cytometry analysis. All Data were analyzed with a FlowJo 6 software.

### Mitochondrial and cytosolic fractionation

Mitochondrial and cytosolic proteins were isolated using the Mitochondria/Cytosol Fractionation kit (Beyotime) according to the manufacturer’s protocol. 2E7 cells were harvested and washed with ice-cold PBS, incubated with 1 mL cytosol extraction buffer mix provided in the kit for 10 min, and then homogenized using ice-cold glass homogenizer. The homogenates were centrifuged at 3,500 × g for 10 min, and the supernatants were further centrifuged at 11,000 × g for 30 min at 4°C. The cytosolic supernatants were decanted, and the pellets resuspended in 1 mL mitochondrial extraction buffer mix. Protein concentrations were determined using the BCA protein quantification assay with BSA as the protein standard.

### Cell transfection

Plasmid of pHA-Rv0928 or pFLAG-Rv0928 was transfected into HEK293T cells with PEI (Polysciences) as previously reported (Grover et al., 2018). To silence SLC25A3 gene expression, siRNA oligonucleotides targeting SLC25A3 (RIBOBIO) were transfected into RAW264.7 cells using Lipomax (Thermo Scientific) according to the manufacturer’s instructions, and the random sequence siRNA oligonucleotides were used as a negative control.

To construct Rv0928-stably expressing RAW264.7 cells, the full-length of Rv0928 gene was ligated into a Plvx-IRES-ZsGreen (pVector) retroviral vector to generate Plvx-IRES-ZsGreen-Rv0928 (pRv0928). HEK293T cells were co-transfected with pspax2 and pVSVG with pRv0928 or pVector using PEI, 48 h later, generated Rv0928-encoding lentiviruses or control lentiviruses were harvested from cell supernatants with ultracentrifugation at 24,000 rpm for 2 h and filtered with a 0.45 μm filter. RAW264.7 cells were infected with Rv0928-encoding lentiviruses or control lentiviruses for 48 h and then directly sorted based on GFP expression with flow cytometry to obtain the Rv0928-stably expressing RAW264.7 cells (RAW-Rv0928) and the control RAW-Vector cells, which were further confirmed by western blot assays for Rv0928 expression.

### Fluorescence and confocal microscopy

After infection with Ms.::Rv0928 or Ms.::Vector for 48 h, apoptotic RAW264.7 cells were detected with One Step TUNEL Apoptosis Assay kit (Beyotime). Cells were fixed with 4% paraformaldehyde, permeabilized with 0.3% Triton-X-100, and then incubated with TdT enzyme and red fluorescence-labeled dUTPs at 37°C for 1 h. After staining nucleus with DAPI (Beyotime) for 10 min at room temperature, the fluorescence-labeled DNA breaks were observed by fluorescence microscopy.

After transfection with pHA-Rv0928 or pFlag-Rv0928 for 48 h, HEK293T cells were fixed with 4% paraformaldehyde, permeabilized with 0.1% Triton X-100 in PBS. Then the cells were then blocked with 3% bovine serum albumin (BSA, Sigma), and incubated with antibody against FLAG, HA, COXIV, GM130, LAMP1, Calnexin and EEA1, respectively, at 4°C overnight. After thoroughly washing, cells were then incubated with DyLight 488-labled goat anti-mouse IgG or DyLight 555-labled goat anti-mouse IgG anti-rabbit IgG (Jackson) for 1 h. After staining nucleus with Hochest 33,342 (Solarbio) for 10 min at room temperature, cells were observed with a laser scanning confocal microscope (Nikon A1).

To detect the localization of cytochrome c or Bax, RAW264.7 cells infected with Ms.::Rv0928 or Ms.::Vector for 48 h, and then cells were incubated in pre-warmed medium containing 250 nM of Mitotracker Red CMXRos (Invitrogen) for 30 min. After washing, cells were fixed using 4% paraformaldehyde, permeabilized with 0.1% Triton X-100 in PBS for 10 min, and then blocked using 3% BSA in PBS; next, they were incubated with anti-cytochrome c or anti-Bax rabbit antibody at 4°C overnight followed by incubation with an Alexa 488-conjugated secondary antibody (Jackson) in the dark for 1 h. After staining nuclei with DAPI for 10 min at room temperature, cells were subjected to confocal microscopy assays.

### Colony-forming unit evaluation

RAW264.7 cells and BMDMs were infected with Ms.::Rv0928 or Ms.::Vector (MOI = 20) for 4 h and then cultured for indicated time periods with 10% FBS-DMEM medium containing 200 μg/mL of amikacin for extracellular bacteria clearance. Then, cells were lysed in 1 mL water plus 0.05% Triton X-100, and 100 μL of serially diluted lysates were incubated to 7H10 agar plates and colony numbers were determined and compared.

Mice were intranasally infected with Ms.::Vector or Ms.::Rv0928 at a dose of 2 × 10^7^ CFU in 30 μL PBS. Six days later, whole lung tissues were collected, weighted, homogenized and suspended in 1 mL PBS. Bacterial loads in lung tissues were measured by plating 100 μL serially diluted homogenates in triplicates on 7H10 agar plates. Colony numbers were determined and compared.

### Assessment of mitochondrial transmembrane potential

Mitochondrial transmembrane potential was assessed by flow cytometry assays using the lipophilic cationic dye JC-1. In cells with high ΔΨm, JC-1 aggregates in mitochondria and emits red fluorescence, while in cells with low ΔΨm, JC-1 remains in monomers and emits green fluorescence. Therefore, the change in JC-1 fluorescence emission would reflect the cell ΔΨm changes. After mycobacterium infection, RAW264.7 cells were incubated with JC-1 dye (Beyotime) at a final concentration of 2 μM for 20 min in the dark. After thoroughly rinsing with PBS, JC-1 fluorescence was monitored by fluorescence microscopy.

### RNA extraction and real-time PCR

RAW264.7 cells were pretreated with 10 μM mitoquinone (Mce) for 30 min, and then infected with Ms.::Vector/Ms.::Rv0928 for 12 h following which the cells were collected and the total RNA was extracted using Trizol reagent (Takara) according to manufacturer’s instructions. The purity and the concentration of the RNA were determined using a Microplate reader (NanoDrop 2000, Wilmington, DE, United States). Total RNA (800 ng) was reverse transcribed using a PrimeScript^®^ RT Reagent Kit with gDNA Eraser (Vazyme). The cDNA was used for quantitative real time PCR (qPCR) analysis on Applied Biosystems 7,500 (Applied Biosystems) with a SYBR^®^ Green PCR Kit (Vazyme). All samples were analyzed in triplicate. The mRNA levels were normalized to those of GAPDH of the same cDNA sample. Relative quantification of gene expression was calculated using the 2^−ΔΔCt^ method.

### Statistical analysis

Data were represented as mean ± standard deviation (SD). GraphPad Prism 7 was used for statistical analysis. Normality was tested by Shapiro–Wilk test. Comparison between 2 groups were performed by Student’s *t*-test analysis. Comparison for more than 2 groups were performed by ANOVA analysis. A *p* < 0.05 was considered significant.

## Results

### Mycobacterial phosphate-binding protein Rv0928 potently impacted the mycobacterium survival in macrophages *in vitro* and *in vivo*

**Figure 1:**
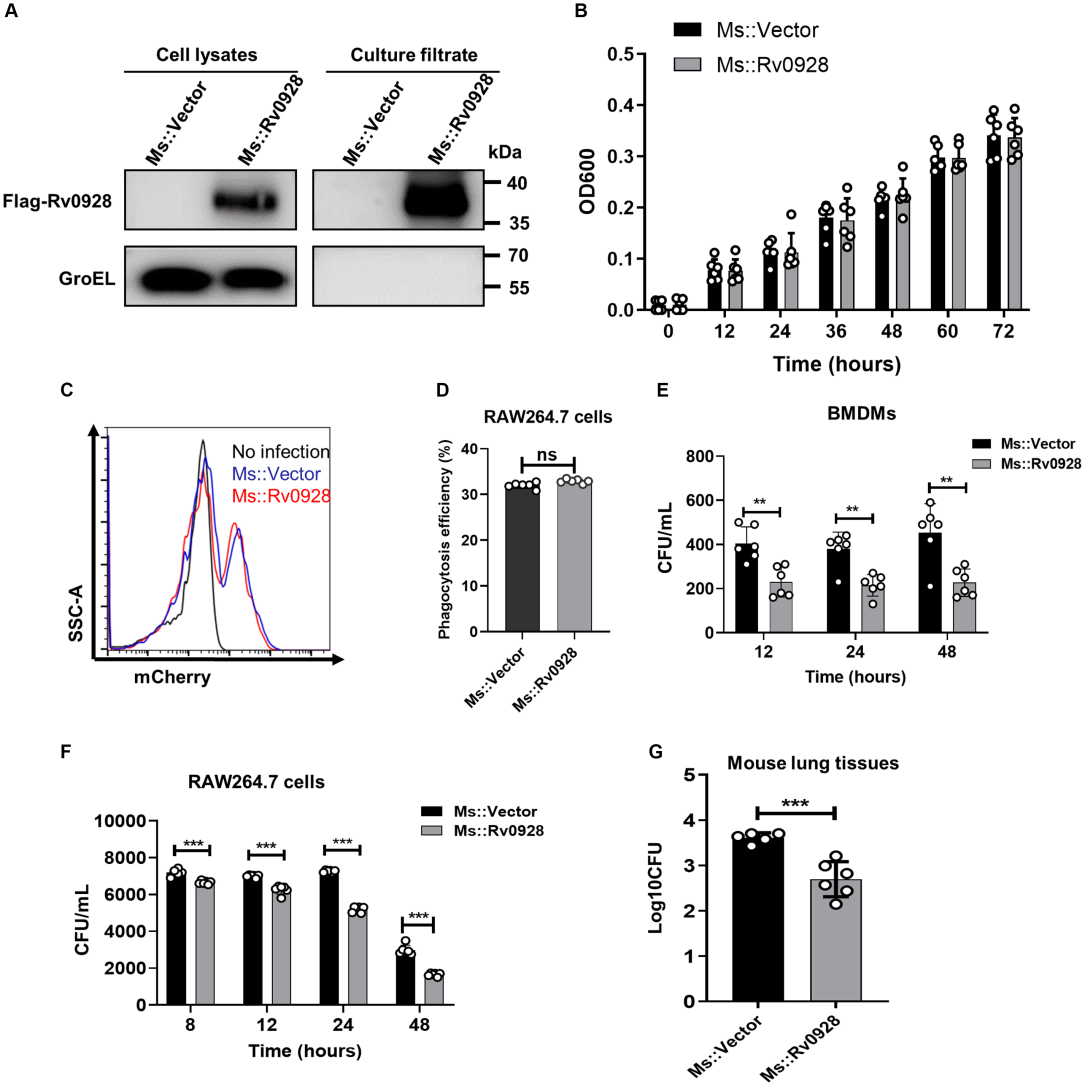
Rv0928 inhibited mycobacterial survival *in vitro* and *in vivo*. **(A)** Culture filtrates and bacterial pellets of Ms.::Vector and Ms.::Rv0928 were subjected to Western blot assays to determine the expression of Flag-tagged Rv0928 protein in the recombinant strain. GroEL was blotted as a marker of bacterial pellets. **(B)** Growth rates of Ms.::Rv0928 and control Ms.::Vector strains in Middlebrook 7H9 broth media. Experiments were repeated 6 times. **(C)** RAW264.7 cells were infected with Ms.::Rv0928-mCherry or Ms.::Vector-mCherry for 2 h and then subjected to flow cytometry assays to detect the phagocytosis capacity. **(D)** Statistical analysis of **(C)**. **(E,F)** Bacterial loads in Ms.::Rv0928-or Ms.::Vector-infected murine BMDMs **(E)** or RAW264.7 cells **(F)** at indicated time points after infection. **(G)** Mice were intranasally infected with Ms.::Vector or Ms.::Rv0928 for 6 days, and then bacterial loads in the lungs were determined by CFU assays. Data are presented as mean ± SD of three independent experiments. **p < 0.01; ***p < 0.001.

To analyze the effects of Rv0928 on mycobacterial infection, a Rv0928-overexpressing Ms. strain (Ms::Rv0928) was constructed and confirmed. Our results showed that Rv0928 was detectable in both the bacterial pellets and culture filtrates of Ms.::Rv0928 (Figure 1A), and that there was no significant difference in the extracellular growth rate in culture media when compared to the control Ms.::Vector strain (Figure 1B). These results suggest that overexpression of Rv0928 did not appear to affect extracellular replication of mycobacteria. Besides, Ms.::Vector and Ms.::Rv0928 carrying mCherry fluorescence were also constructed and used to infect RAW264.7 cells. we collected the cells at 2 h after infection, and performed flow assays to assess the ability of macrophages to phagocytose these two recombinant Ms. strains. The results showed that macrophage RAW264.7 cells possessed similar phagocytosis capacity for these two Mycobacterium strains (Figures 1C,D). In contrast, the intracellular loads of Ms.::Rv0928 in infected murine BMDMs (Figure 1E), as well as murine macrophage RAW264.7 cells (Figure 1F) were significantly lower than those of Ms.::Vector-infected cells at the indicated time points (8, 12, 24, and 48 h post infection). Similarly reduced bacterial loads were also detected in the lungs of Ms.::Rv0928-infected mice, with the log value of colony-forming unit (CFU) reaching approximately 2.7, significantly lower than 3.6 in Ms.::Vector-infected mice (Figure 1G), indicating that Rv0928 obviously suppressed the mycobacterial survival both *in vitro* and *in vivo*.

### Rv0928 overexpression promoted caspase-9-mediated intrinsic apoptosis of mycobacterium-infected macrophages

**Figure 2:**
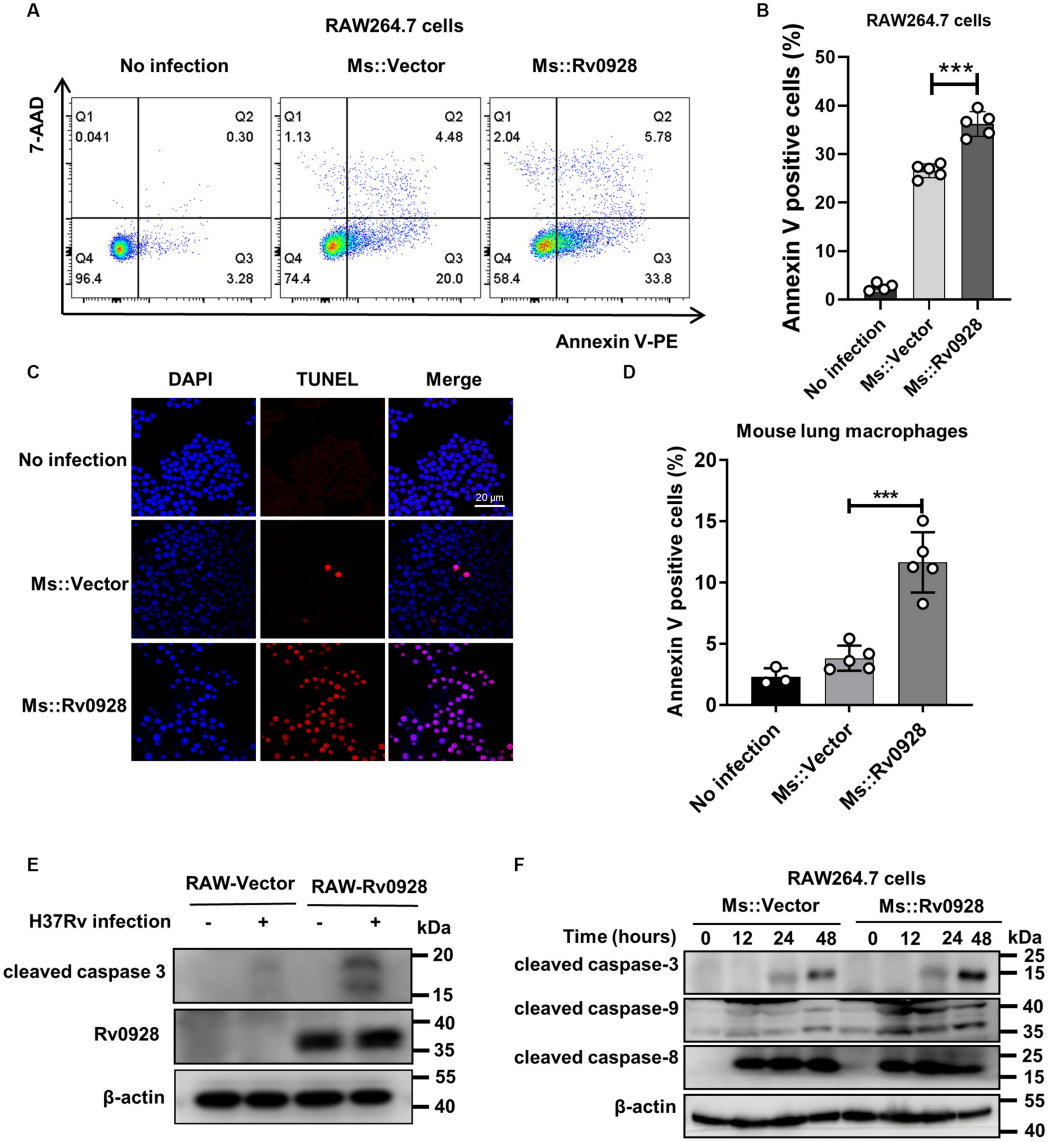
Rv0928 increased caspase-9-mediated intrinsic apoptosis in mycobacterium-infected macrophages. **(A)** RAW264.7 cells were infected with Ms.::Rv0928 or Ms.::Vector for 48 h and then subjected to flow cytometry assays to detect the frequency of apoptotic cells. **(B)** Statistical analysis of **(A)**. **(C)** RAW264.7 cells were infected with Ms.::Rv0928 or Ms.::Vector for 48 h and then cell apoptosis was detected by TUNEL assay. **(D)** Mice were infected with Ms.::Rv0928 or Ms.::Vector for 6 days, and then the Annexin V^+^ apoptotic lung macrophages (CD11b^+^F4/80^+^) were detected by flow cytometry assays. Each group contained 5 mice. **(E)** RAW-Vector or RAW-Rv0928 cells were infected with H37Rv for 48 h, and then the cleaved caspase-3 protein was determined by western blot assay. **(F)** In addition, the expression of cleaved caspase-8, caspase-9 and caspase-3 in Ms-infected RAW264.7 cells were detected by western blot assays at indicated time points. Data are representative of two independent experiments. ***p < 0.001.

We found that compared to the control Ms.::Vector strain, Ms.::Rv0928 infection induced much higher percentages of apoptosis in murine RAW264.7cells (Figures 2A–C), as well as murine primary CD11b^+^F4/80^+^ lung macrophages (Figure 2D), as detected by flow cytometry assays or TUNEL assays. Meanwhile, we observed that the cleaved caspase-3 expression was significantly increased in Rv0928-stably expressing RAW264.7 cells (RAW-Rv0928) compared to RAW-Vector cells post H37Rv infection, indicating a higher activation of a pro-apoptotic pathway (Figure 2E). In addition, the expression of cleaved caspase-9 (an intrinsic apoptosis initiator) and cleaved caspase-3 (a terminal apoptosis executor) were obviously increased in Ms.::Rv0928 infected RAW264.7 cells, whereas the expression of cleaved caspase-8 (an extrinsic apoptosis initiator) was hardly changed in both infected RAW264.7 cells (Figure 2F). These data indicated that mycobacterial Rv0928 promoted the intrinsic apoptosis in infected macrophages.

### Mycobacterium Rv0928 augmented the inflammatory responses of infected macrophages by facilitating NF-κB and MAPK activation

**Figure 3:**
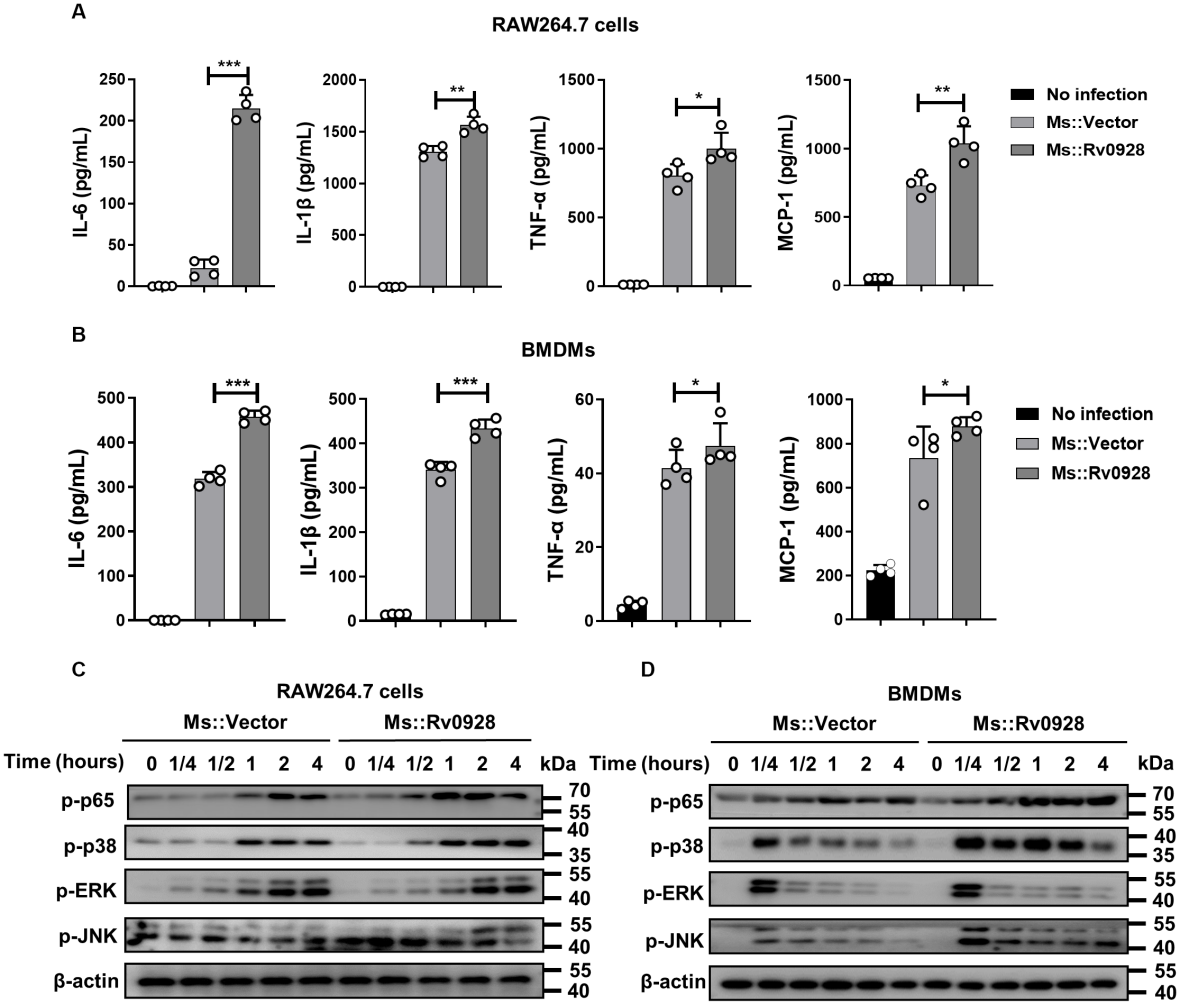
Rv0928 enhanced inflammatory responses in mycobacterium-infected macrophages. RAW264.7 cells **(A)** and BMDMs **(B)** were infected with Ms.::Rv0928 or Ms.::Vector for 12 h, and then the levels of pro-inflammatory cytokines (IL-6, IL-1β, TNF-a, and MCP-1) in cell supernatants were determined by ELISA assays. Data are presented as mean ± SD of three independent experiments. *p < 0.05; **p < 0.01; ***p < 0.001. Meanwhile, the expression of phosphorylated p65, p38, JNK, and ERK in infected RAW264.7 cells **(C)** and BMDMs **(D)** were also detected by Western blot assays. The experiment was repeated independently 3 times with similar results.

Apart from the increased apoptosis, we noticed that Ms.::Rv0928 infection also elicited more robust productions of inflammatory cytokines in infected RAW264.7 cells. The levels of IL-6, TNF-α, IL-1β as well as MCP-1 were achieved 235, 1,171, 1,568 and 1,197 pg./mL, respectively, significantly higher than those in Ms.::Vector-infected cells (31, 696, 1,263, and 749 pg./mL, respectively) (Figure 3A). Similar phenomenon was also evidenced in infected BMDMs (Figure 3B). To better understand the signaling pathways initiated by Ms.::Rv0928, the phosphorylation status of inflammatory key factors NF-kB subunit p65, p38, ERK, and JNK was detected by western blot assays. We found that Rv0928 over-expression could obviously increase the phosphorylation of p65, p38 as well as JNK in infected macrophages, while no obvious change was seen in ERK phosphorylation (Figures 3C,D), indicating that Rv0928 mainly amplified mycobacterium-induced inflammation via increasing NF-κB and MAPK pathways.

### Mycobacterial Rv0928 localized to the mitochondrion and interacted with the mitochondrial phosphate carrier SLC25A3 protein

**Figure 4:**
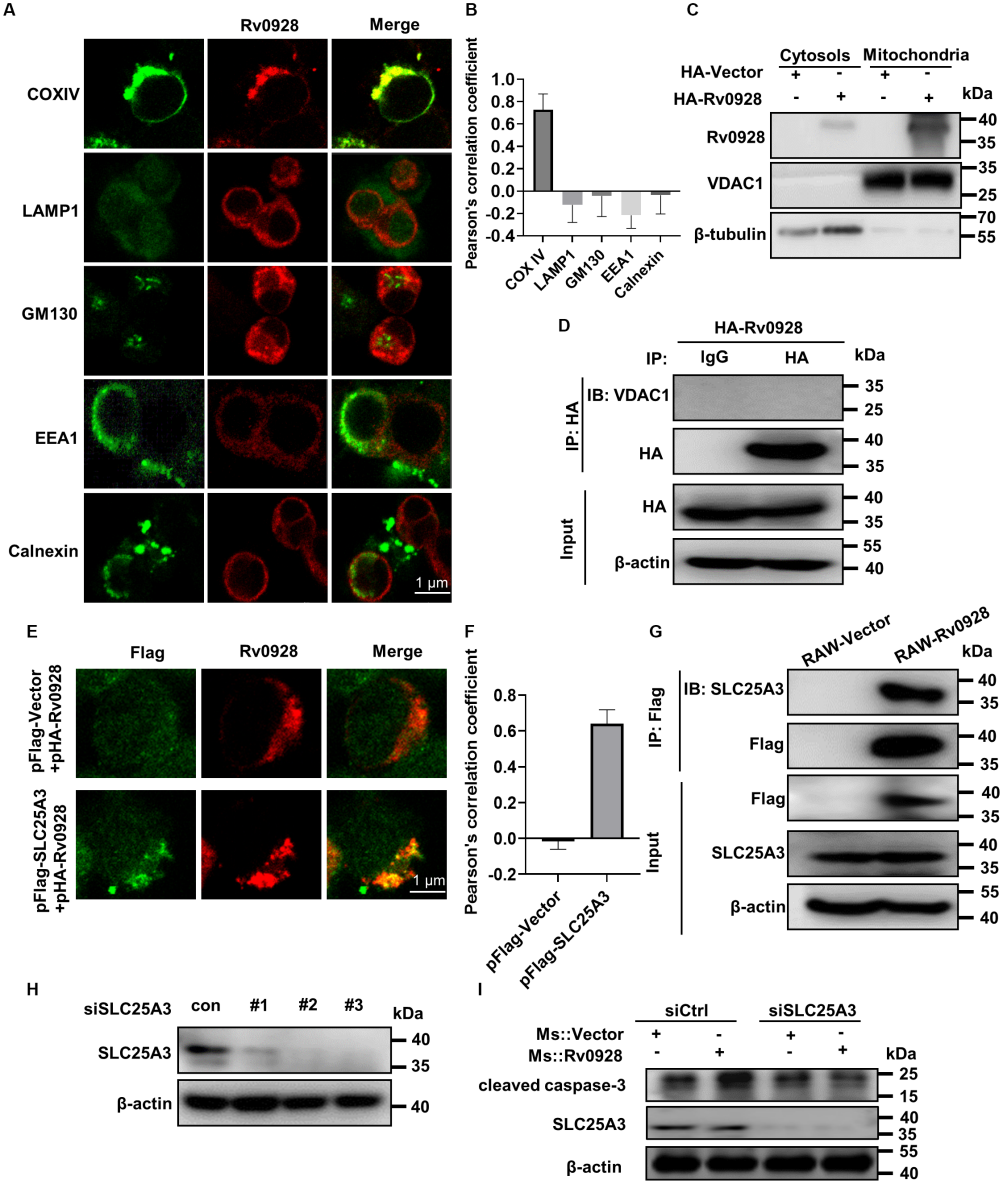
Rv0928 co-localized with SLC25A3 protein in mitochondria. **(A)** HEK293T cells were transfected with pFlag-Rv0928 or pHA-Rv0928 for 48 h, and then the co-localization of Rv0928 protein with various organelle markers (COX IV for mitochondria, LAMP1 for lysosome, GM130 for Golgi apparatus, EEA1 for early endosome, Calnexin for endoplasmic reticulum) was observed by confocal assays. **(B)** Pearson correlation coefficient of **(A)**, and the results are given by image J analysis software (*n* = 20). **(C)** HEK293T cells were transfected with pHA-Rv0928 for 48 h, and the Rv0928 expression in cytosols and mitochondria was detected by Western blot assays. **(D)** Co-immunoprecipitation of Rv0928 and mitochondrial VDAC1 protein. HEK293T cells were transfected with pHA-Rv0928 for 48 h, and then cell extracts were immunoprecipitated with anti-HA-beads, followed by immunoblotting with anti-VDAC1 or anti-HA antibody. **(E)** HEK293T cells were co-transfected with pHA-Rv0928 and pFlag-SLC25A3 or pFlag-Vector for 48 h, and co-localization of Rv0928 with mitochondrial SLC25A3 was observed by confocal assays. **(F)** Pearson correlation coefficient of **(E)**. **(G)** Co-immunoprecipitation of Rv0928 and mitochondrial phosphate carrier SLC25A3 protein. RAW264.7 cells stably expressing Flag-Rv0928 were harvested, and cell extracts were immunoprecipitated with anti-Flag beads and analyzed with the indicated antibodies. **(H)** The RAW264.7 cells were transfected with three SLC25A3 specific siRNAs in concentration 100 nM. 48 h post transfection the SLC25A3 protein level was detected by western blotting. **(I)** After transfection with SLC25A3-specific or control siRNA for 24 h, RAW264.7 cells were infected with Ms.::Rv0928 or Ms.::Vector for 48 h, and then the expression of cleaved caspase-3 was detected by Western blot. The experiments were repeated 3 times independently with similar results.

Identifying the subcellular localization of proteins is crucial for elucidating their biological functions and molecular mechanisms. Interestingly, immunofluorescence analysis of exogenously expressed Rv0928 in HEK293T cells revealed a staining pattern that specifically overlapped with the mitochondrial marker COX IV, rather than GM130^+^ Golgi apparatus, LAMP1^+^ lysosome, EEA1^+^ early endosome or Calnexin^+^ endoplasmic reticulum (Figures 4A,B). This observation was further supported by the Western blot assays, which identified a substantial amount of Rv0928 protein was identified in purified mitochondria, but not in cytosols (Figure 4C). Previous studies have demonstrated the co-localization of various pathogen proteins with mitochondria by interacting with mitochondrial membrane proteins, such as voltage-dependent anion channels (VDACs) (Massari et al., 2000; Rahmani et al., 2000; Han et al., 2019), but here we did not detect the interaction of Rv0928 with VDAC1 by co-immunoprecipitation assays (Figure 4D). Indeed, interactions between bacterial and host proteins belonging to the same superfamily have been reported to assist the bacterial protein to co-localize in mitochondria and affect mitochondrial stability. For example, Mtb-derived heat shock protein (HSP) 65 could interact with and stabilize the mitochondrial mortalin protein, a member of the host HSP70 family, and then inhibit PAPR-mediated macrophage apoptosis (Joseph et al., 2017). Therefore, we hypothesized that Rv0928 as a mycobacterial phosphate transporter, might interact with the host phosphate carrier SLC25A3, which located on the mitochondrial inner membrane (Seifert et al., 2015). As expected, we observed an obvious co-localization of Rv0928 with SLC25A3 in co-transfected HEK293T cells by immunofluorescence assays (Figures 4E,F), and further confirmed the interaction of these two proteins by co-immunoprecipitation assays in RAW-Rv0928 cells (Figure 4G). To determine the relationship SLC25A3 with Rv0928-induced cell apoptosis, we first downregulated SLC25A3 expression in RAW264.7 cells via siRNA. RAW264.7 cells were individually transfected with three siRNAs targeting different sites, and SLC25A3 protein expression was measured 48 h post-transfection. It was found that the 2 # and 3 # siRNA obviously reduced the SLC25A3 level relative to the siRNA control (Figure 4H). More importantly, targeted silencing of SLC25A3 expression with siRNA significantly reduced Rv0928-induced caspase-3 activation in RAW264.7 cells (Figure 4I), further confirming that SLC25A3 was at least partially involved in Rv0928-mediated macrophage apoptosis.

### Rv0928 altered mitochondrial membrane potential and permeabilization, and promoted cytochrome c release into cytosols

**Figure 5:**
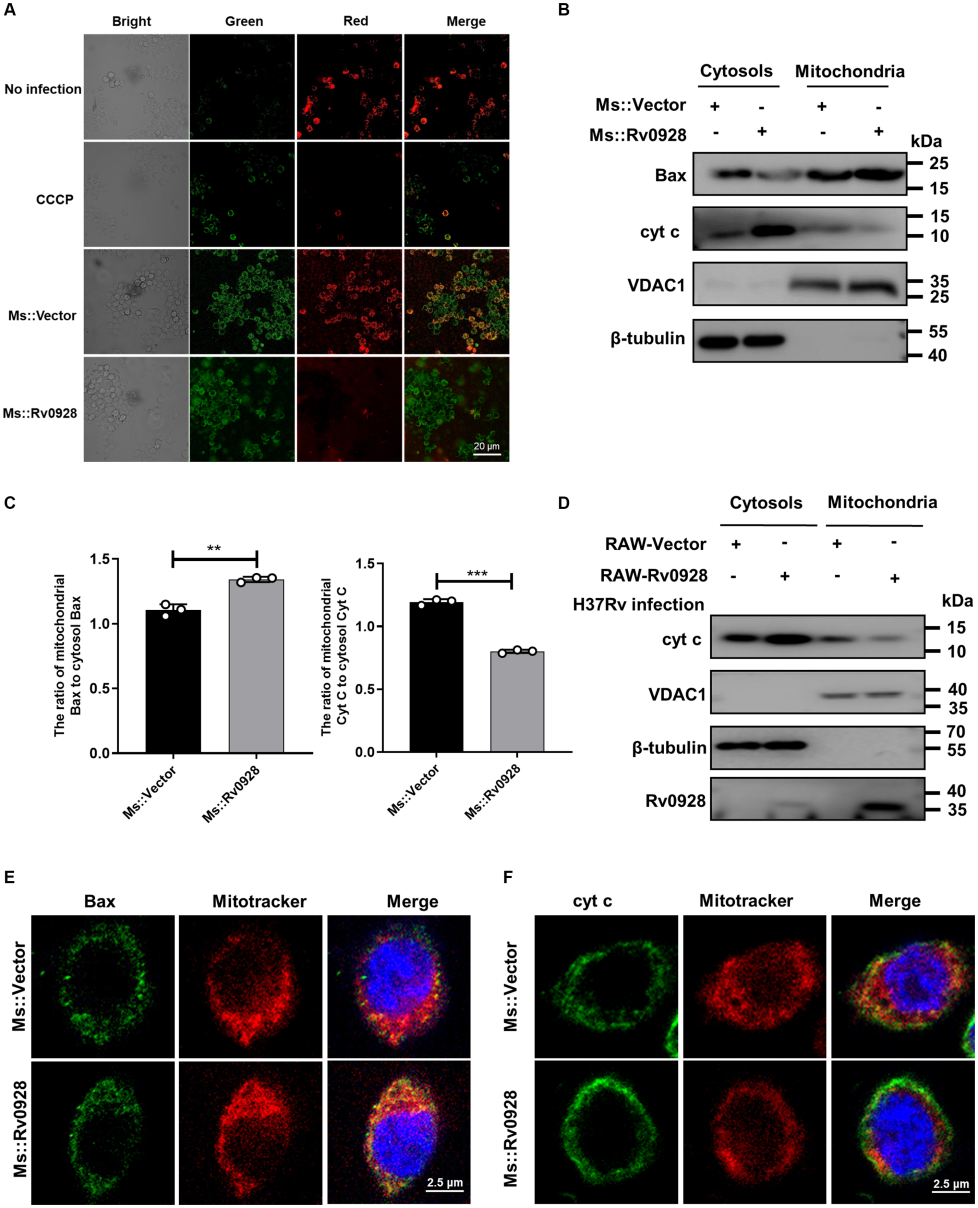
Mycobacterial Rv0928 caused mitochondrial membrane depolarization in infected macrophages. **(A)** The mitochondrial membrane potentials (ΔΨm) of Ms.::Rv0928-or Ms.::Vector-infected RAW264.7 cells were evaluated with the fluorescence changes of JC-1 dye using fluorescence microscopy, where the fluorescence switching from red to green indicates a loss of ΔΨm. **(B)** RAW264.7 cells were infected with Ms.::Rv0928 or Ms.::Vector for 48 h. Mitochondrial and cytosolic fractions were separated, and the levels of Bax and cytochrome c (cyt c) were detected by Western blot assays. VDAC1 and β-tubulin were used as markers for mitochondrial and cytosolic fractions, respectively. **(C)** Statistical analysis of **(B)**. **p < 0.01; ***p < 0.001. Data are presented as mean ± SD of three independent experiments. **(D)** RAW-Vector or RAW-Rv0928 cells were infected with H37Rv for 48 h, Mitochondrial and cytosolic fractions were separated, and the levels of cytochrome c (cyt c) were detected by Western blot assays. VDAC1 and β-tubulin were used as markers of mitochondrial and cytosolic fractions, respectively. Meanwhile, the mitochondrial localization of Bax **(E)** or cytochrome c **(F)** in Ms.::Rv0928 infected RAW264.7 cells was also observed by confocal assays. The experiments were repeated 3 times independently with similar results.

We observed that the mitochondrial membrane potentials were decreased in Ms.::Rv0928-infected macrophages, as reflected by the decreased red fluorescence of aggregated JC-1 dye in mitochondria and the increased green fluorescence of monomeric JC-1 dye in cytosols (Figure 5A), suggesting that Rv0928 tended to accumulate in mitochondria and disrupted their homeostasis by depolarizing the membrane potentials of infected macrophages. Mitochondrial membrane depolarization (loss of ΔΨm) has been reported to correlate closely with apoptotic cell death by causing mitochondrial outer membrane permeabilization (MOMP) (Duan et al., 2002). Consistently, we observed that Ms.::Rv0928 infection caused greater accumulation of mitochondrial Bax and cytosolic cytochrome c in macrophages compared to those infected with Ms.::Vector (Figures 5B,C). In addition, overexpression of Rv0928 significantly promoted the release of mitochondrial cytochrome c into cytosols in H37Rv-infected cells (Figure 5D). To further validate the translocation of Bax and cytochrome c induced by Rv0928, confocal assays were performed to detect the subcellular localization of these two proteins. The results shown in Figure 5E revealed a significant increase in the co-localization of Bax protein with the mitotracker-stained mitochondria in cells infected with Ms.::Rv0928 compared to those infected with Ms.::Vector. Conversely, the co-localization of cytochrome c with mitochondria was significantly reduced (Figure 5F). Collectively, these results provide compelling evidence that Rv0928 enhanced the mycobacterium-induced translocation of Bax, the master effector of MOMP, from the cytosol into the mitochondria, which in turn induce the mitochondrial cytochrome c release into the cytoplasm, ultimately triggering caspase-9-mediated mitochondrial apoptosis of Ms.::Rv0928-infected macrophages (Figure 2).

### Rv0928 amplified mycobacterium-induced macrophage inflammation by increasing mitochondrial reactive oxygen species production

**Figure 6:**
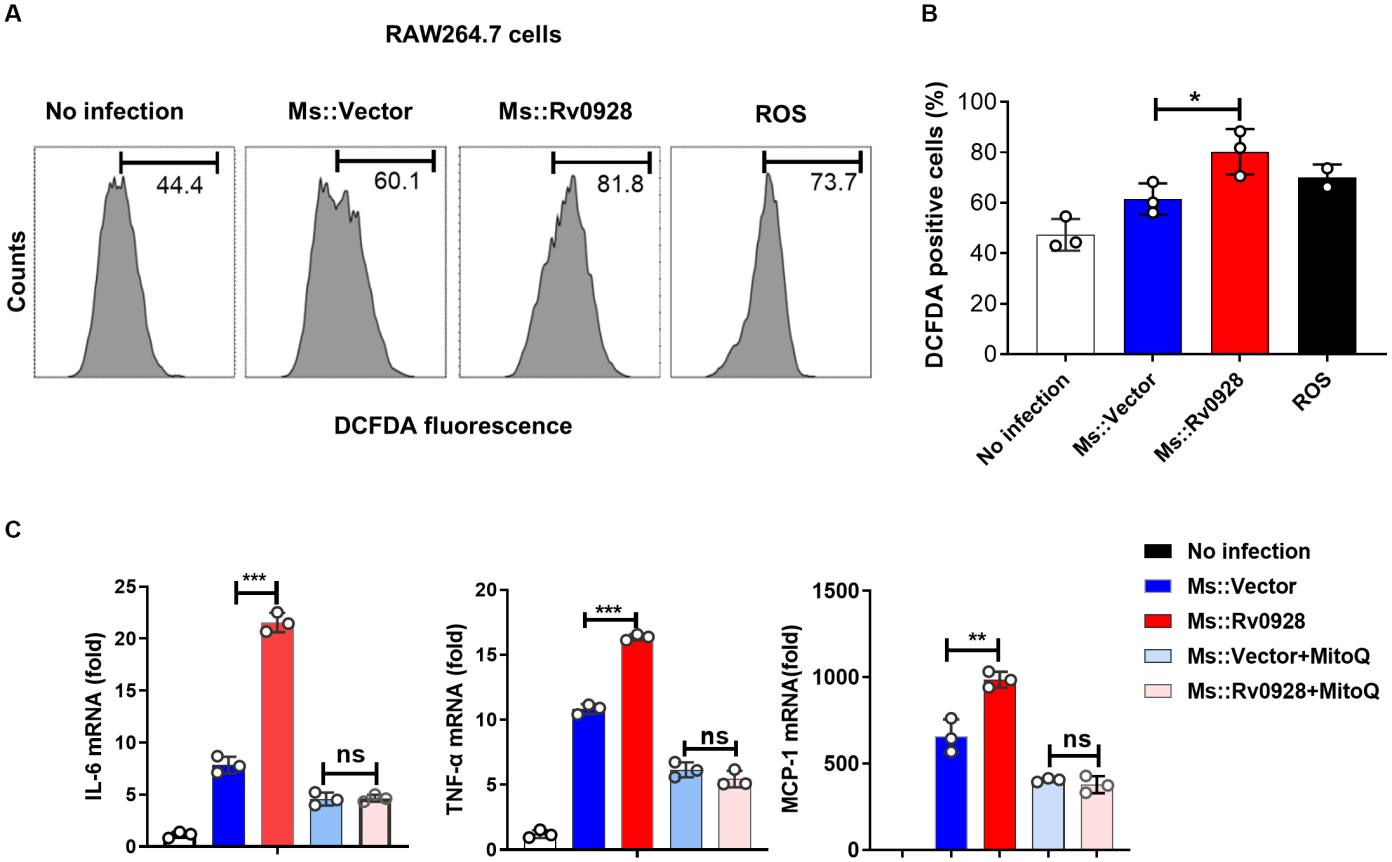
Rv0928 augmented inflammatory responses of infected macrophages by enhancing mitochondrial ROS production **(A)** ROS in Ms.::Rv0928-infected RAW264.7 cells were stained with DCFDA dye and monitored by flow cytometry assays. **(B)** Statistical analysis of **(A)**. **(C)** RAW264.7 cells were infected with Ms.::Rv0928 or Ms.::Vector in the presence of mitochondria ROS inhibitor MitoQ for 12 h, and then the mRNA level of pro-inflammatory cytokines were monitored by RT-qPCR analysis. *p < 0.05; **p < 0.01; ***p < 0.001. Data were shown as mean ± SD of three independent experiments.

**Figure 7:**
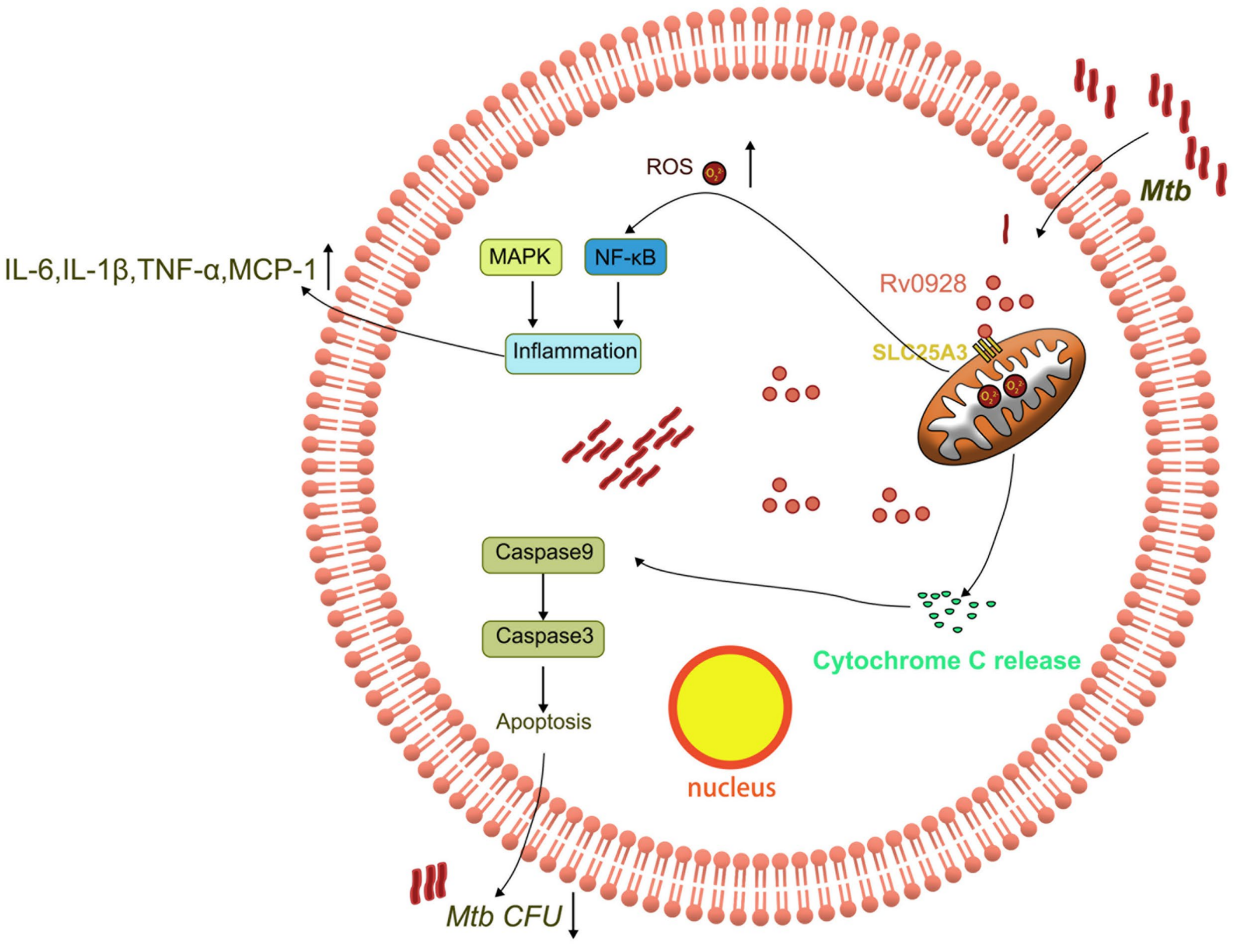
Schematic representation of the mechanism by which Rv0928 facilitated macrophage clearance of Mtb infection by affecting mitochondrion-associated apoptosis and inflammation.

ΔΨm depolarization has been documented to increase the production of reactive oxygen species (ROS), which subsequently triggers immune activation and inflammation (Wang et al., 2009; Bulua et al., 2011; Park et al., 2015). In this study, we found that Ms.::Rv0928 induced a higher level of ROS production versus the control Ms.::Vector strain, as reflected by a much higher percentage of H_2_DCFDA^+^ (a ROS fluorescent probe) in infected macrophages (81.8% vs. 60.1%, *p* < 0.05, Figures 6A,B). Notably, the mitochondrial ROS scavenger scavenger mitoquinone (MitoQ) significantly reversed the pro-inflammation effects of Rv0928 macrophages (Figure 6C). These data indicated that the Rv0928-induced disruption of mitochondrial homeostasis promoted the ROS production, which in turn enhanced macrophage inflammation by promoting NF-κB and MAPK pathways (Figure 7).

## Discussion

Increasing evidence has shown that Pst system proteins also possess other biological functions beside of trans-membrane transport of phosphate. For example, Mtb extracellular vesicle formation was recently reported relating to Pst system (White et al., 2018). PstS1 and Rv0928 protein vaccines were shown to induce Th1 and Th17 responses in mice although not conferring protection against Mtb infection (Palma et al., 2011). Therefore, herein we explored the impact of Rv0928 on the interaction of Mtb and macrophages as well as its role in intracellular Mtb infection and survivals.

We found that recombinant Ms.::Rv0928 strain showed a similar growth rate to the control Ms.::Vector in phosphate-sufficient media (containing 25 mM Pi), but showed a significantly reduced survival in infected macrophages. We further demonstrated that Ms.::Rv0928 infection significantly increased macrophage apoptosis as well as proinflammatory cytokine production, indicated that Rv0928 altered the cellular biology and immune function of infected macrophages. In consistent with our observation, Dasgupta et al. (2010) also reported that another Mtb ABC transporter Rv1280c-Rv1283c system could also regulate cytokine release and apoptosis of infected macrophages via its substrate-binding polypeptide OppA, although its role in intracellular bacterial survival was not explored.

We further found that Rv0928 mainly accumulated in mitochondria and caused the mitochondrial membrane potential depolarization and permeabilization alteration. This led to the release of mitochondrial cyt c into cytosols to unleash the capsapse-9-meidated intrinsic apoptotic cascade pathway. Actually, in addition to PstS3 protein, other PstS proteins like PstS1 have also been found to significantly promote the apoptosis of Mtb-infected macrophages, but with different molecular mechanisms, like triggering Fas–FasL extrinsic apoptosis pathway (Parandhaman and Narayanan, 2014). Our study further expanded the pro-apoptosis mechanism of PstS proteins on macrophages to the intrinsic mitochondrial pathway. Meanwhile, we also noticed that the perturbed mitochondrial membrane potentials by Rv0928 also amplified the mitochondrial ROS production as well as the subsequent activation of redox-sensitive transcription factor NF-κB as well as MAPK inflammatory pathways.

So far, we are still unclear about the accumulation mechanism of Rv0928 protein in macrophage mitochondria. Previous studies showed that Mtb secretory proteins mainly entry into the cytoplasm via the Mtb ESAT-6-or PDIM-forming pores on phagosomal membranes (Peng and Sun, 2016), and then bacterial proteins frequently associate with mitochondria by the following three mechanisms: Firstly, proteins contain N-terminal mitochondrial targeting sequences which guide their translocation to mitochondria, while no such sequences were predicted in Rv0928 protein when we used the TargetP website. Secondly, some proteins with β-barrel structures may be associated with mitochondrial by integrating into the mitochondrial membrane or other undefined mechanisms. However, no such β-barrel structures were evidenced in Rv0928, either (Ferraris et al., 2014). Thirdly, other bacterial proteins that do not belong to either above-mentioned category. Accordingly, we roughly estimated that Rv0928 protein might be assigned to the third category.

SLC25A3 protein, a member of solute carrier family 25, locates on the inner membrane of mitochondria and is mainly responsible for transporting Pi into the mitochondrial matrix. Recently, a serious of literatures demonstrated a key modulating effect of SLC25A3 on mitochondrial membrane potentials (Leung et al., 2008), mitochondrial stability (Kwong et al., 2014), and mitochondrion-associated cell death (Büttner et al., 2011). In addition, SLC25A3 also seems to represent a relatively common mitochondrial protein which responses to infection of various pathogens, including cytome-galovirus (CMV) (Pauleau et al., 2008), transmissible gastroenteritis virus (TGEV) (Guo et al., 2022). Consistently, herein we have found that SLC25A3 was involved in the Mtb infection. Meanwhile, whether Rv0928 had an effect on the expression and function of SLC25A3 protein was also unknown. Further studies need to address these issues in depth.

It is well-accepted that Mtb infection of macrophages is a subtle, complicated and dynamic host-pathogen interaction with varied outcomes depending on the nature of the Mtb strain and immune status of the host (Kolloli and Subbian, 2017; Lam et al., 2017; Liu et al., 2017; Mahamed et al., 2017). However, in the late granuloma stage, macrophage apoptosis and inflammation would facilitate Mtb dissemination (Davis and Ramakrishnan, 2009; Pagán and Ramakrishnan, 2014). As for the Rv0928-enhanced macrophage apoptosis and inflammation, we still lacked solid evidence to show whether it was actively initiated by mycobacteria to facilitate its dissemination *in vivo*, or whether it was a host immune-enhancing mechanism to control the intracellular survival of the Rv0928-upregulated mycobacteria. However, considering the significantly decreased pulmonary mycobacterial loads in Ms.::Rv0928-infected mice, we tend to assume that this enhanced apoptosis and inflammation elicited by Rv0928 protein was more likely to be a host immune-enhancing strategy, in which macrophages actively adapted to and control the infection of mycobacteria with the induced Rv0928 system. Of course, this hypothesis needs to be further validated.

## Data availability statement

The original contributions presented in the study are included in the article. Further inquiries can be directed to the corresponding author.

## Ethics statement

Ethical approval was not required for the studies on humans in accordance with the local legislation and institutional requirements because only commercially available established cell lines were used. The animal study was approved by the Institutional Animal Care and Use Committees of Soochow University. The study was conducted in accordance with the local legislation and institutional requirements.

## Author contributions

CX: Data curation, Investigation, Writing – original draft. YY: Supervision, Validation, Writing – review & editing. SX: Funding acquisition, Resources, Supervision, Writing – review & editing.

## References

[ref1] Bulua A. C. Simon A. Maddipati R. Pelletier M. Park H. Kim K. Y.. (2011). Mitochondrial reactive oxygen species promote production of proinflammatory cytokines and are elevated in TNFR1-associated periodic syndrome (TRAPS). J. Exp. Med. 208, 519–533. doi: 10.1084/jem.20102049, PMID: 21282379 PMC3058571

[ref2] Büttner S. Ruli D. Vögtle F. N. Galluzzi L. Moitzi B. Eisenberg T.. (2011). A yeast BH3-only protein mediates the mitochondrial pathway of apoptosis. EMBO J. 30, 2779–2792. doi: 10.1038/emboj.2011.197, PMID: 21673659 PMC3160254

[ref3] Dasgupta A. Sureka K. Mitra D. Saha B. Sanyal S. Das A. K.. (2010). An oligopeptide transporter of *Mycobacterium tuberculosis* regulates cytokine release and apoptosis of infected macrophages. PLoS One 5:e12225. doi: 10.1371/journal.pone.0012225, PMID: 20808924 PMC2923189

[ref4] Davis J. M. Ramakrishnan L. (2009). The role of the granuloma in expansion and dissemination of early tuberculous infection. Cells 136, 37–49. doi: 10.1016/j.cell.2008.11.014, PMID: 19135887 PMC3134310

[ref5] Duan L. Gan H. Golan D. E. Remold H. G. (2002). Critical role of mitochondrial damage in determining outcome of macrophage infection with *Mycobacterium tuberculosis*. J. Immunol. 169, 5181–5187. doi: 10.4049/jimmunol.169.9.5181, PMID: 12391235

[ref6] Ferraris D. M. Spallek R. Oehlmann W. Singh M. Rizzi M. (2014). Crystal structure of the *Mycobacterium tuberculosis* phosphate binding protein PstS3. Proteins 82, 2268–2274. doi: 10.1002/prot.24548, PMID: 24615888

[ref7] Grover S. Sharma T. Singh Y. Kohli S. P M. Singh A.. (2018). The PGRS domain of *Mycobacterium tuberculosis* PE_PGRS protein Rv0297 is involved in endoplasmic reticulum stress-mediated apoptosis through toll-like receptor 4. MBio 9:e01017-18. doi: 10.1128/mBio.01017-18, PMID: 29921671 PMC6016250

[ref8] Guo J. Liu Z. Zhang D. Lai Y. Gao J. Wang X.. (2022). circEZH2 inhibits opening of mitochondrial permeability transition pore via interacting with PiC and up-regulating RSAD2. Vet. Microbiol. 272:109497. doi: 10.1016/j.vetmic.2022.109497, PMID: 35785658

[ref9] Han B. Ma Y. Tu V. Tomita T. Mayoral J. Williams T.. (2019). Microsporidia interact with host cell mitochondria via voltage-dependent anion channels using Sporoplasm surface protein 1. MBio 10:e01944-19. doi: 10.1128/mBio.01944-19 31431557 PMC6703431

[ref10] Hegarty P. K. Sfakianos J. P. Giannarini G. DiNardo A. R. Kamat A. M. (2020). COVID-19 and Bacillus Calmette-Guérin: what is the link? Eur. Urol. Oncol. 3, 259–261. doi: 10.1016/j.euo.2020.04.001, PMID: 32327396 PMC7152883

[ref11] Joseph S. Yuen A. Singh V. Hmama Z. (2017). *Mycobacterium tuberculosis* Cpn60.2 (GroEL2) blocks macrophage apoptosis via interaction with mitochondrial mortalin. Biol Open 6, 481–488. doi: 10.1242/bio.023119, PMID: 28288970 PMC5399554

[ref12] Kolloli A. Subbian S. (2017). Host-directed therapeutic strategies for tuberculosis. Front. Med. 4:171. doi: 10.3389/fmed.2017.00171 PMC5651239 29094039

[ref13] Kwong J. Q. Davis J. Baines C. P. Sargent M. A. Karch J. Wang X.. (2014). Genetic deletion of the mitochondrial phosphate carrier desensitizes the mitochondrial permeability transition pore and causes cardiomyopathy. Cell Death Differ. 21, 1209–1217. doi: 10.1038/cdd.2014.36, PMID: 24658400 PMC4085527

[ref14] Lam A. Prabhu R. Gross C. M. Riesenberg L. A. Singh V. Aggarwal S. (2017). Role of apoptosis and autophagy in tuberculosis. Am. J. Physiol. Lung Cell. Mol. Physiol. 313, L218–l229. doi: 10.1152/ajplung.00162.2017, PMID: 28495854 PMC5582934

[ref15] Lamarche M. G. Wanner B. L. Crépin S. Harel J. (2008). The phosphate regulon and bacterial virulence: a regulatory network connecting phosphate homeostasis and pathogenesis. FEMS Microbiol. Rev. 32, 461–473. doi: 10.1111/j.1574-6976.2008.00101.x, PMID: 18248418

[ref16] Leung A. W. Varanyuwatana P. Halestrap A. P. (2008). The mitochondrial phosphate carrier interacts with cyclophilin D and may play a key role in the permeability transition. J. Biol. Chem. 283, 26312–26323. doi: 10.1074/jbc.M805235200, PMID: 18667415 PMC3258905

[ref17] Liu C. H. Liu H. Ge B. (2017). Innate immunity in tuberculosis: host defense vs pathogen evasion. Cell. Mol. Immunol. 14, 963–975. doi: 10.1038/cmi.2017.88, PMID: 28890547 PMC5719146

[ref18] Mahamed D. Boulle M. Ganga Y. Mc Arthur C. Skroch S. Oom L.. (2017). Intracellular growth of *Mycobacterium tuberculosis* after macrophage cell death leads to serial killing of host cells. Elife 6:e22028. doi: 10.7554/eLife.28205, PMID: 28130921 PMC5319838

[ref19] Massari P. Ho Y. Wetzler L. M. (2000). *Neisseria meningitidis* porin PorB interacts with mitochondria and protects cells from apoptosis. Proc. Natl. Acad. Sci. U. S. A. 97, 9070–9075. doi: 10.1073/pnas.97.16.9070, PMID: 10922061 PMC16823

[ref20] Nieuwenhuizen N. E. Kaufmann S. H. E. (2018). Next-generation vaccines based on Bacille Calmette-Guérin. Front. Immunol. 9:121. doi: 10.3389/fimmu.2018.00121 29459859 PMC5807593

[ref21] Pagán A. J. Ramakrishnan L. (2014). Immunity and immunopathology in the tuberculous granuloma. Cold Spring Harb. Perspect. Med. 5:a018499. doi: 10.1101/cshperspect.a018499 25377142 PMC4561401

[ref22] Palma C. Spallek R. Piccaro G. Pardini M. Jonas F. Oehlmann W.. (2011). The *M. tuberculosis* phosphate-binding lipoproteins PstS1 and PstS3 induce Th1 and Th17 responses that are not associated with protection against *M. tuberculosis* infection. Clin. Dev. Immunol. 2011:690328. doi: 10.1155/2011/690328, PMID: 21603219 PMC3095447

[ref23] Parandhaman D. K. Narayanan S. (2014). Cell death paradigms in the pathogenesis of *Mycobacterium tuberculosis* infection. Front. Cell. Infect. Microbiol. 4:31. doi: 10.3389/fcimb.2014.00031, PMID: 24634891 PMC3943388

[ref24] Park J. Min J. S. Kim B. Chae U. B. Yun J. W. Choi M. S.. (2015). Mitochondrial ROS govern the LPS-induced pro-inflammatory response in microglia cells by regulating MAPK and NF-κB pathways. Neurosci. Lett. 584, 191–196. doi: 10.1016/j.neulet.2014.10.016, PMID: 25459294

[ref25] Pauleau A. L. Galluzzi L. Scholz S. R. Larochette N. Kepp O. Kroemer G. (2008). Unexpected role of the phosphate carrier in mitochondrial fragmentation. Cell Death Differ. 15, 616–618. doi: 10.1038/sj.cdd.4402295, PMID: 18174900

[ref26] Peirs P. Lefèvre P. Boarbi S. Wang X. M. Denis O. Braibant M.. (2005). *Mycobacterium tuberculosis* with disruption in genes encoding the phosphate binding proteins PstS1 and PstS2 is deficient in phosphate uptake and demonstrates reduced *in vivo* virulence. Infect. Immun. 73, 1898–1902. doi: 10.1128/IAI.73.3.1898-1902.2005, PMID: 15731097 PMC1064925

[ref27] Peng X. Sun J. (2016). Mechanism of ESAT-6 membrane interaction and its roles in pathogenesis of *Mycobacterium tuberculosis*. Toxicon 116, 29–34. doi: 10.1016/j.toxicon.2015.10.003, PMID: 26456678 PMC4973572

[ref28] Rahmani Z. Huh K. W. Lasher R. Siddiqui A. (2000). Hepatitis B virus X protein colocalizes to mitochondria with a human voltage-dependent anion channel, HVDAC3, and alters its transmembrane potential. J. Virol. 74, 2840–2846. doi: 10.1128/JVI.74.6.2840-2846.2000, PMID: 10684300 PMC111774

[ref29] Rengarajan J. Bloom B. R. Rubin E. J. (2005). Genome-wide requirements for *Mycobacterium tuberculosis* adaptation and survival in macrophages. Proc. Natl. Acad. Sci. U. S. A. 102, 8327–8332. doi: 10.1073/pnas.0503272102, PMID: 15928073 PMC1142121

[ref30] Rifat D. Bishai W. R. Karakousis P. C. (2009). Phosphate depletion: a novel trigger for *Mycobacterium tuberculosis* persistence. J. Infect. Dis. 200, 1126–1135. doi: 10.1086/605700, PMID: 19686042

[ref31] Seifert E. L. Ligeti E. Mayr J. A. Sondheimer N. Hajnóczky G. (2015). The mitochondrial phosphate carrier: role in oxidative metabolism, calcium handling and mitochondrial disease. Biochem. Biophys. Res. Commun. 464, 369–375. doi: 10.1016/j.bbrc.2015.06.031, PMID: 26091567 PMC8011645

[ref32] Singh S. Saavedra-Avila N. A. Tiwari S. Porcelli S. A. (2022). A century of BCG vaccination: immune mechanisms, animal models, non-traditional routes and implications for COVID-19. Front. Immunol. 13:959656. doi: 10.3389/fimmu.2022.959656, PMID: 36091032 PMC9459386

[ref33] Soni D. K. Dubey S. K. Bhatnagar R. (2020). ATP-binding cassette (ABC) import systems of *Mycobacterium tuberculosis*: target for drug and vaccine development. Emerg. Microbes Infect. 9, 207–220. doi: 10.1080/22221751.2020.1714488, PMID: 31985348 PMC7034087

[ref34] Vanzembergh F. Peirs P. Lefevre P. Celio N. Mathys V. Content J.. (2010). Effect of PstS sub-units or PknD deficiency on the survival of *Mycobacterium tuberculosis*. Tuberculosis 90, 338–345. doi: 10.1016/j.tube.2010.09.004, PMID: 20933472

[ref35] Wang C. C. Fang K. M. Yang C. S. Tzeng S. F. (2009). Reactive oxygen species-induced cell death of rat primary astrocytes through mitochondria-mediated mechanism. J. Cell. Biochem. 107, 933–943. doi: 10.1002/jcb.22196, PMID: 19459161

[ref36] White D. W. Elliott S. R. Odean E. Bemis L. T. Tischler A. D. (2018). *Mycobacterium tuberculosis* Pst/SenX3-RegX3 regulates membrane vesicle production independently of ESX-5 activity. MBio 9:e00778-18. doi: 10.1128/mBio.00778-18, PMID: 29895636 PMC6016242

[ref37] World Health Organization (2022) Global tuberculosis report 2022. Available at: www.who.int/teams/global-tuberculosis-programme/tb-reports/global-tuberculosis-report-2022.

[ref38] Zhang X. Goncalves R. Mosser D. M. (2008). The isolation and characterization of murine macrophages. Curr. Protoc. Immunol. 14, 14.1.1–14.1.14. doi: 10.1002/0471142735.im1401s83 PMC2834554 19016445

